# Association of circulating levels of MMP-8 with mortality from respiratory disease in patients with rheumatoid arthritis

**DOI:** 10.1186/ar4042

**Published:** 2012-10-02

**Authors:** Derek L Mattey, Nicola B Nixon, Peter T Dawes

**Affiliations:** 1 Haywood Rheumatology Centre, University Hospital of North Staffordshire, Staffordshire, England, ST6 7AG, UK; 2 Institute of Science and Technology in Medicine, Keele University, Staffordshire, England, ST5 5BG, UK

## Abstract

**Introduction:**

Matrix metalloproteinases (MMPs) are implicated in the destruction of the joint and have been shown to be strongly associated with inflammation in rheumatoid arthritis (RA). Circulating MMPs have also been associated with cardiovascular disease in the general population, and are predictive of cardiovascular mortality. The purpose of the present study was to determine whether circulating levels of MMPs are predictive of mortality in RA.

**Methods:**

A multiplex suspension array system (Luminex^®^) was used to measure levels of MMPs (1, 2, 3, 8 and 9) in sera taken at recruitment of RA patients (*n *= 487) in a study of factors associated with mortality in RA. Patients were tracked on the National Health Service Central Register for notification of death, and the relationship between baseline MMP levels and mortality was analysed using Cox proportional hazards regression analysis.

**Results:**

At the time of follow-up, 204/486 patients had died, of which 94 (46.1%) had died of circulatory diseases, 49 of malignancy (24.0%), and 42 (20.6%) of respiratory diseases. In a stepwise analysis which included all MMPs, only MMP-8 was significantly associated with all cause mortality (*P *= 0.0007, 0.6% hazard ratio increase per ng/ml). No association was found between MMP levels and mortality due to circulatory disease or malignancy. However MMP-8 levels were strongly associated with mortality due to respiratory disease (*P *< 0.0001, 1.3% hazard ratio increase per ng/ml). The association with respiratory disease related mortality remained highly significant in multivariate models which included smoking as well as markers of severity and disease activity such as rheumatoid factor, nodular disease, and C-reactive protein (CRP).

**Conclusions:**

The serum level of MMP-8 is a strong predictor of mortality in RA, especially that due to respiratory disease. This finding is consistent with increased activation of neutrophils in RA and identifies serum MMP-8 as a useful marker for increased risk of premature death.

## Introduction

Many studies have shown that the mortality rate is increased in patients with rheumatoid arthritis (RA) compared with the general population [1-6]. Causes of death in RA are similar to those in the general population but there is an increased risk of death due to cardiovascular disease (CVD), and an excess of deaths due to infection, much of which is due to lower respiratory tract infections [1,2,7-12].

Markers of inflammation such as erythrocyte sedimentation rate (ESR), C-reactive protein (CRP) and soluble tumour necrosis factor receptors have been shown to be predictive of mortality in RA [2,3,5,7-9,13], but many other markers associated with the disease process have not been investigated. Some of these may provide alternative or better predictors of early mortality. One such group of markers are the matrix metalloproteinases (MMPs). These are implicated in the destruction of the joint and have been shown to be strongly associated with inflammation and disease activity in RA [14-18]. Circulating MMPs have also been associated with CVD and atherosclerotic plaque instability in non-RA patients [19-25], and have been shown to be predictive of cardiovascular mortality [20,25].

We postulated that elevated levels of circulating MMPs in RA patients may be associated not only with increased rheumatoid disease activity and severity, but may also predict premature mortality. We have thus investigated the relationship of circulating levels of MMPs with all-cause, and cause-specific mortality, and determined whether any relationship between mortality and MMP levels was independent of traditional risk factors and other markers of inflammation and disease severity.

## Materials and methods

### Study population

This was a follow up study of a cohort of RA patients from North Staffordshire, England, recruited between 1993 and 1998 to investigate long-term outcome and mortality in patients attending a hospital-based clinic at the Haywood Rheumatology Centre. Consecutive cases were selected from clinics of confirmed RA patients who satisfied the 1987 American College of Rheumatology criteria for RA [26]. Baseline assessments included the disability index of the Stanford health assessment questionnaire (HAQ) [27], presence of erosions, CRP and ESR levels, IgM rheumatoid factor (RF), and presence or absence of nodular disease. Age, sex, disease duration at recruitment, and history of current or past cigarette smoking was also recorded. As an indication of pre-existing CVD at the time of recruitment, patients were stratified according to whether or not they were taking any drugs for cardiovascular problems. This included patients taking drugs for cardiac and non-cardiac conditions, the latter including cerebrovascular disease and peripheral vascular disease. Patients taking drugs for hypertension alone were not included in this group. The study was approved by the North Staffordshire local research ethics committee.

The treatment of patients at baseline reflected standard UK practice for management of hospital-based RA patients as outlined in published guidelines [28]. Patients were receiving anti-inflammatory and/or anti-rheumatic therapy, with the majority of patients (> 90%) being treated with one or more disease modifying anti-rheumatic drugs (DMARDs). DMARDs were chosen according to physician's preference, using the standard practice at the time of sequential monotherapy and combination therapy for more severe disease. The most common combination was methotrexate and sulphasalazine. Steroids and cytotoxic drugs such as azathioprine or cyclophosphamide were received by a small minority of individuals (< 5%). A number of patients (8%) were treated with anti-TNF agents during the latter years of the study (from 2002). These patients all fulfilled the United Kingdom National Institute of Clinical Excellence (NICE) criteria for use of anti-TNF therapy.

### Survival follow up

All patients were registered on the NHS Central Register (NHSCR), a computerised registry of the records of all patients registered with a general practitioner in England and Wales. Access to this registry was obtained via the Office for National Statistics (ONS), General Register Office, Southport, UK. Patients were tracked on the NHSCR, and notification of patient deaths was obtained from the ONS within 1 month of death. Causes of death were coded by the ONS, using the International Classification of Diseases Ninth Revision (ICD-9) [29] up until 31 December 2000, and ICD-10 after this date. Patients were followed up to 31 December 2011.

### Multiplex MMP assays

Sera were separated from bloods collected in plain glass Becton Dickinson (BD) (Becton Dickinson, Oxford, Oxfordshire, UK) Vacutainer^® ^tubes at study entry. Bloods were allowed to clot for 60 minutes at room temperature before serum separation and storage at -70^o ^C until required. Measurement of MMP-1, 2, 3, 8 and 9 levels was performed on the serum samples using Fluorokine MAP multiplex kits (R&D Systems, Minneapolis, MN, USA) and was read on a Luminex^® ^suspension array system (Bio-Plex 200™, Bio-Rad (Bio-Rad Laboratories, Hemel Hempstead, Hertfordshire, UK)). Sample preparation and assay procedure were followed according to the manufacturer's recommendation. Heteroblock (Omega Biologicals, Bozeman, MT, USA) was added at a concentration of 150 ug/ml into the sample diluent prior to assay, to block any non-specific binding to RF [30]. All samples were run in duplicate with the appropriate standards on 96-well microplates. The limit of detection for each of the MMPs was as follows: MMP-1, 0.04 ng/ml; MMP-2, 0.03 ng/ml; MMP-3, 0.01 ng/ml; MMP-8, 0.04 ng/ml; MMP-9, 0.74 ng/ml. The intra- and inter-assay coefficients of variation for each of the MMPs were between 1.5 and 9.2%, and 6.2 and 15.2% respectively.

### Statistical analysis

Spearman's rank correlation was used to assess the relationship between MMP levels and measures of disease activity and severity. The Mann-Whitney *U*-test was used to determine baseline differences in MMP levels between patients with and without nodular or erosive disease, between smokers and non-smokers and between surviving and non-surviving patients. The association between serum MMP levels and mortality risk was investigated using Cox proportional hazard regression analyses adjusted for age, sex and disease duration at baseline. The time intervals for those patients who were alive at the end of the study period and those who were lost to follow up were censored. The censoring date was the date of the last hospital visit. Since mortality data are recorded centrally in the UK, we were able to collect mortality data on all patients entered into the study, and patients lost to follow up without a documented death could be censored with some degree of certainty. Multivariate stepwise models were used to assess the predictive value of MMP levels compared with other potential baseline risk factors (such as, RF, nodules, CRP, ESR, HAQ, taking CVD drugs, smoking status). Separate analyses were carried out on the risk of mortality related to each of the major causes of death in RA, namely circulatory, malignant and respiratory diseases. In these analyses the data on subjects who died of other causes, and the data on those who were still alive at last follow up were censored.

Kaplan-Meier curves were plotted to illustrate survival in relation to median MMP levels at baseline. All data were analysed using Number Cruncher Statistical Software package for Windows (NCSS 2000, NCSS Statistical Software, Kaysville, UT, USA), and GraphPad Prism software (version 1.03, GraphPad Software Incorporated, San Diego, CA, USA).

## Results

### Characteristics of RA patients

The baseline clinical features of the cohort are summarised in Table 1. Of the 487 patients recruited there were 204 deaths (41.9%), of which 86 were men and 118 were women. For patients who died the median survival time from study baseline was 6.9 years (interquartile range (IQR) 3.6 to 12.1). For survivors the median follow up period from baseline was 16.2 years (IQR 5.9 to 17.2).

**Table 1:** Characteristics of rheumatoid arthritis (RA) patients at baseline

Number	487
Male/female	194/293
Age (SD)*	58.3 (12.5)
Age of onset (SD)*	48.9 (13.0)
Duration, years (SD)*	9.4 (8.3)
Rheumatoid factor positive (%)	283 (58.1)
Nodules (%)	84 (17.2)
Erosive (%)	405 (83.2)
HAQ (IQR)	1.625 (1.0, 2.125)

*Mean values and standard deviation (SD). HAQ, Health Assessment Questionnaire; IQR, interquartile range.

### Main causes of death

The three major causes of death were due to circulatory disease (94/204, 46.1%), neoplasia (49/204, 24.0%) and respiratory diseases (42/204, 20.6%). Of the 94 patients who died from circulatory disease, 62 were due to heart disease (mainly ischaemic heart disease, congestive heart failure, or left ventricular failure), and 19 were due to cerebrovascular disease. The main cause of respiratory disease related mortality was pneumonia (29/42). In this group, pneumonia was recorded as the only cause of death, or the only cause with RA as a contributing cause in 23/29 patients. Of the remaining six patients with pneumonia, three also had longstanding chronic obstructive pulmonary disease (COPD), one had pulmonary fibrosis, one had CVD and one was associated with sepsis.

### Relationship between MMP levels and patient characteristics at baseline

There was a positive correlation between the levels of all the MMPs apart from MMP-2 (Additional file 1). Comparison of MMP levels in men and women showed a significantly higher median level of MMP-3 (28.1 v 21.2 ng/ml, *P *= 0.00008), MMP-8 (23.37 v 17.96 ng/ml, *P *= 0.0008) and MMP-9 (435.5 v 363.5 ng/ml, *P *= 0.001) in men. The levels of MMP-1, MMP-2 and MMP-3 showed a significant increase with age (R_s _≥ 0.106, *P *≤ 0.01), independent of disease duration (Additional file 2). Most measures of disease activity and severity (ESR, CRP, HAQ, Larsen score, visual analogue (VAS) pain score) were positively correlated with levels of all MMPs apart from MMP-2 (Additional file 2). The latter showed a negative correlation with CRP and the VAS pain score. No correlation was found between IgM RF titres and MMP levels. Patients with erosive disease had significantly higher levels of MMP-3 than those with non-erosive disease (25.46 v 15.35 ng/ml, *P *= 0.006), but no difference was found for other MMPs. The association with MMP-3 remained significant after adjusting for age, sex and disease duration (data not shown). No difference in MMP levels was found between patients with or without nodular disease. Patients who were taking one or more drugs for CVD at baseline had significantly higher levels of MMP-2, MMP-8 and MMP-9 than patients not taking these drugs (146.3 v 131.1 ng/ml, *P *= 0.007, 26.67 v 20.32 ng/ml, *P *= 0.02, and 471.5 v 413.7, *P *= 0.049 respectively). However, in a stepwise analysis adjusted for age, sex and disease duration, there was only an association with MMP-9 levels (data not shown). Patients that had ever smoked also had higher levels of MMP-8 and MMP-9 than those who had never smoked (22.05 v 17.46 ng/ml, *P *= 0.015, and 411.2 v 344.8 ng/ml, *P *= 0.004 respectively).

### Relationship between MMP levels and mortality

Compared with surviving patients, baseline levels of MMP-2 were significantly higher in patients who died during follow up (Table 2). Stratification by causes of death also demonstrated that higher levels of MMP-2 were found in patients who later died from circulatory disease, but higher levels of MMP-8 were found in patients who died from respiratory disease. Baseline MMP-1 levels were lower in patients who later died from malignant disease, but this association disappeared after adjusting for age, sex and disease duration (data not shown). The association of high baseline MMP-2 levels with patients who later died also disappeared after adjusting for age, sex and disease duration. However, the association of high MMP-8 levels with patients later dying of respiratory disease remained after such adjustment (*P *= 0.01).

**Table 2:** Circulating baseline levels of metalloproteinases (MMPs) in surviving and non-surviving rheumatoid arthritis patients

	Survivors	Non survivors by major causes of death			
		**All causes**	**Circulatory disease**	**Neoplastic disease**	**Respiratory disease**

Number	283	204	94	49	42
MMP-1	3.35	3.60	3.42	2.52	4.93
MMP-2	129.09	141.11	150.08	137.51	139.73
MMP-3	23.22	27.42	25.63	27.89	27.42
MMP-8	20.03	20.50	18.67	18.55	25.16*
MMP-9	384.81	375.82	377.44	386.38	449.75

Serum levels of MMPs (ng/ml) are medians.*P (adjusted for age, sex and disease duration) = 0.01 (compared with surviving patients).

We next investigated whether the mortality risk increased with increasing levels of MMPs. Separate Cox analysis for each MMP, with adjustment for age, sex and disease duration demonstrated significant increases in the hazard ratio (HR) with increasing levels of MMP-8 and MMP-9 (Table 3). No association with MMP-2 levels was seen in Cox models adjusted for age. Analyses of cause-specific mortality demonstrated significant associations of MMP-8 and MMP-9 with mortality due to respiratory disease (Table 4). No associations were found with any MMPs for deaths due to circulatory disease or neoplasia.

**Table 3:** Association of baseline levels of individual metalloproteinaises (MMPs) with all-cause mortality

MMP (ng/ml)	Hazard ratio (95% CI)	*P*
MMP-1	1.003 (0.99, 1.01)	0.3
MMP-2	1.0005 (0.99, 1.001)	0.7
MMP-3	0.999 (0.998, 1.000)	0.07
MMP-8	1.006 (1.002, 1.01)	0.003
MMP-9	1.005 (1.00, 1.001)	0.04

Separate Cox proportional hazard regression analyses were carried out for each MMP with adjustment for age, sex and disease duration at baseline.

**Table 4:** Association of baseline levels of individual metalloproteinases (MMPs) with respiratory disease-related mortality

MMP (ng/ml)	Hazard ratio (95% CI)	*P*
MMP-1	1.007 (0.99, 1.001)	0.1
MMP-2	1.000 (0.99, 1.000)	1.0
MMP-3	0.997 (0.994, 0.999)	0.3
MMP-8	1.013 (1.005, 1.02)	< 0.0001
MMP-9	1.002 (1.00, 1.004)	0.002

Separate Cox proportional hazard regression analyses were carried out for each MMP with adjustment for age, sex and disease duration at baseline.

### Multivariate survival analysis

In stepwise Cox proportional hazards regression models that included all MMPs, only MMP-8 was found to be significantly associated with all-cause mortality (HR = 1.006 per ng/ml, 95% CI 1.002, 1.01, *P *= 0.003). Further multivariate analyses including other risk factors revealed that MMP-8 level was predictive of mortality independently of age, smoking at baseline, nodular disease, and taking CVD drugs at baseline, all of which were associated with all-cause mortality (Table 5). Other disease activity and severity markers (CRP, ESR, HAQ, RF, Larsen score) were excluded due to non-significance in stepwise analysis (Table 5).

**Table 5:** Multivariate baseline predictors of all-cause and respiratory disease-related mortality in rheumatoid arthritis patients

Step and variable	Hazard ratio (95% CI)	*P*
All-cause mortality		

1. Age (years)	1.08 (1.06, 1.09)	< 0.0001
2. Nodular disease	2.05 (1.27, 3.31)	< 0.0001
3. Taking CVD drugs	1.96 (1.25, 3.05)	0.002
4. Smoking at baseline	1.97 (1.25, 3.10)	0.007
5. MMP-8 (ng/ml)	1.01 (1.002, 1.02)	0.01

Respiratory disease mortality		

1. Age (years)	1.12 (1.08, 1.16)	< 0.0001
2. MMP-8 (ng/ml)	1.013 (1.006, 1.02)	< 0.0001
3. Nodular disease	2.76 (1.38, 5.50)	0.0002
4. Rheumatoid factor	2.57 (1.18, 5.58)	0.02

Stepwise Cox proportional hazards regression. Baseline variables included in the analyses were age, sex, disease duration, MMP-8 (ng/ml), rheumatoid factor (+/-), nodular disease (+/-), C-reactive protein (mg/l), erythrocyte sedimentation rate (mm/h), Health Assessment Questionnaire (score 0 to 3), Larsen score, taking drugs for cardiovascular disease (CVD) (+/-) and smoking at entry (+/-). Only significant variables retained in the stepwise analysis are shown.

Mortality due to circulatory diseases was not associated with levels of any MMP, although when mortality due to heart disease alone was analysed a significant association with baseline MMP-9 levels was found (HR 1.0013 per ng/ml, *P *= 0.01). This remained significant (*P *= 0.01) after adjusting for smoking at baseline and nodular disease, but significance was lost when adjusted for patients taking CVD drugs at baseline. The latter association was highly significant (HR 4.60, 95% CI 2.39, 8.94, *P *< 0.0001). As in the case of all-cause mortality, we found that age, smoking at baseline and nodular disease were all significantly associated, with or without adjustment for taking CVD drugs.

Mortality due to neoplasia was not associated with levels of any MMP. The most significant predictors were older age, and smoking at baseline (data not shown). Multivariate analysis of mortality due to respiratory disease showed that MMP-8 level was a highly significant predictor along with age, nodular disease and presence of RF (Table 5). Although significant in univariate analysis, MMP-9 levels were not significantly associated in a model that also included MMP-8 levels. Smoking and measures of inflammation were not associated. Separate analyses of patients who had died from pneumonia or from other causes of respiratory disease demonstrated a significant association with MMP-8 levels in both cases, although the number of patients who had died from causes other than pneumonia was small (*n *= 13) (Table 6).

**Table 6:** Multivariate baseline predictors of mortality due to pneumonia and mortality due to other respiratory diseases in rheumatoid arthritis patients

Step and variable	Hazard ratio (95% CI)	*P*
Mortality due to pneumonia		

1. Age	1.08/yr (1.05, 1.17)	< 0.0001
2. MMP-8 (ng/ml)	1.012 (1.010, 1.025)	0.0002
3. Nodular disease	2.36 (1.01, 5.53)	0.01
4. Rheumatoid factor	2.66 (1.03, 6.82)	0.05

Mortality due to other respiratory diseases*		

1. Age	1.09/yr (1.03, 1.16)	0.006
2. Nodular disease	4.45 (1.55, 12.81)	0.006
3. MMP-8 (ng/ml)	1.016 (1.004, 1.03)	0.004

Stepwise Cox proportional hazards regression. Baseline variables included in the analyses were age, sex, disease duration, metalloproteinase (MMP)-8 (ng/ml), rheumatoid factor (+/-), nodular disease (+/-), C-reactive protein (mg/dl), erythrocyte sedimentation rate (mm/h), Health Assessment Questionnaire (score 0 to 3), Larsen score, taking drugs for cardiovascular disease (CVD) (+/-) and smoking at entry (+/-). Only significant variables retained in the stepwise analysis are shown. *Includes pulmonary fibrosis (*n *= 3), chronic obstructive pulmonary disease (*n *= 4), bronchitis (*n *= 2), bronchiectasis (*n *= 3) and asthma (*n *= 1).

**Figure 1:**
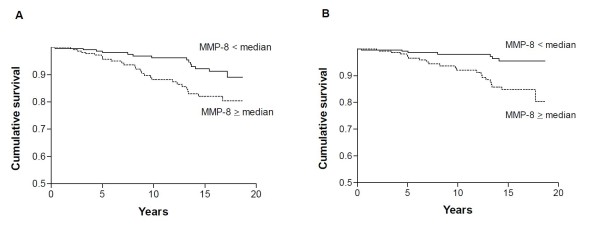
**Kaplan Meier survival curves for RA patients showing the relationship between baseline serum levels of metalloproteinase (MMP)-8 and mortality due to respiratory disease or pneumonia alone**. **(A) **Respiratory disease. (**B**) Pneumonia alone. For these analyses, the levels of MMP-8 were divided into those below (<) or equal to and above (≥) the median (20.07 ng/ml).

Figure 1 illustrates the survival of patients in relation to the median cutoff level for MMP-8 (20.07 ng/ml) at baseline using Kaplan-Meier analysis to generate survival curves based on mortality due to respiratory disease (Figure 1A) or pneumonia alone (Figure 1B). Levels equal to and above the median were associated with significantly worse survival (HR 2.63, 95% CI 1.38, 5.05, *P *= 0.003 and HR 4.44, 95% CI 1.86, 10.59, *P *= 0.0007 respectively, after adjustment for age, sex and disease duration at baseline).

## Discussion

Our data indicate that the serum level of MMP-8 (neutrophil collagenase) is a strong predictor of mortality in RA, especially that due to respiratory disease. As far as we are aware, this is the first study to identify a biomarker that is predictive of respiratory disease-related mortality in RA. Age, nodular disease and presence of RF were also predictive but the MMP-8 association was independent of these. Although the majority of deaths in this group of patients were due to pneumonia, separate cause-specific analyses revealed that MMP-8 levels were associated with mortality from both pneumonia and other forms of respiratory disease. The latter included pulmonary fibrosis, COPD, bronchitis, and bronchiectasis, but these were analysed as a single group since the numbers were too small to investigate specific sub-groups. Further research on larger cohorts of patients will be needed to determine whether MMP-8 levels are predictive of mortality from specific respiratory diseases other than pneumonia.

Apart from MMP-8 and age, the other baseline parameters most strongly associated with mortality in this study were smoking, (all-cause mortality, cancer mortality), taking CVD drugs (all cause-mortality, cardiovascular mortality), nodular disease (all-cause mortality, respiratory disease mortality), and RF (all-cause mortality, respiratory disease mortality). The findings on all-cause mortality are consistent with previous studies in RA. Although we and others have shown that higher levels of MMP-8 are associated with smoking in RA and other conditions [31,32], our results suggest that the association of MMP-8 with all-cause and respiratory disease mortality is independent of smoking.

It is interesting that none of the other MMPs examined here were associated with mortality after adjusting for MMP-8 levels or age, sex and disease duration, even though all of the MMPs, apart from MMP-2, showed significant correlations with inflammatory and disease severity markers, and the literature on non-RA populations has reported associations of MMP-2, MMP-3 and MMP-9 levels with CVD [19-24]. In this study we found that the baseline levels of MMP-2 were significantly higher in patients who subsequently died in the follow up period, but the association disappeared after adjusting for age. This can be explained by the significant association between age and MMP-2 levels seen in the correlation analyses. Although we found no association of MMP levels with mortality from circulatory diseases in general, we did find that MMP-9 levels were associated with mortality specifically due to heart disease. This association disappeared after adjusting for patients taking CVD drugs at baseline. This can probably be explained by our data showing that these particular patients had significantly higher levels of MMP-9, and is consistent with previous studies in no-RA populations showing higher levels of MMP-9 in heart disease [19-21,24].

The mechanisms behind the association of MMP-8 levels with mortality from respiratory disease are likely to involve the tissue destructive effects of this MMP. Several cell types may contribute towards the levels of MMP-8 but the major source is most likely to be activated neutrophils. Many previous studies have demonstrated the likely role of neutrophil-derived MMPs in the pathogenesis of respiratory diseases, including acute respiratory distress syndrome or acute lung injury [33,34], COPD [35,36], cystic fibrosis [37], interstitial lung disease [38], idiopathic pulmonary fibrosis [39] and bronchiectasis [40,41]. Furthermore, pulmonary MMP concentrations have been shown to be elevated in patients with hospital-acquired pneumonia [42,43]. All of these conditions are characterized by an influx of neutrophils into sites of inflammation and subsequent pulmonary tissue injury, the severity of which is associated with MMP levels [39,40,42,43].

It is interesting in this study that measurement of MMP-8 levels at a single time point was predictive of long-term mortality, even though in many cases the levels were measured years before death. One possibility is that elevated MMP-8 levels may reflect chronic ongoing neutrophil activation, which may persist for many years in RA. Peripheral blood neutrophils in RA patients have been reported to be primed but show functional impairment in Fc-mediated generation of reactive oxygen species [44]. This has been suggested to account for the increased susceptibility to bacterial infection in patients with severe RA and may explain the increase in respiratory diseases such as pneumonia. The presence of high-risk bacteria in patients with hospital-acquired pneumonia has been shown to be associated with significantly higher MMP-8 and MMP-9 levels and activity in the bronchoalveolar lavage fluid [42,43]. It has also been reported that artificial ventilation may further promote protease activation [43,45].

There are a number of possible limitations to the present study. Apart from determining which patients were taking CVD drugs, other comorbidities were not recorded at baseline. Various comorbid conditions may be associated with elevated levels of MMPs, so these may represent unmeasured confounders. The information on patients taking drugs for CVD problems allowed us to provide an estimate of patients suffering from cardiovascular morbidity at the time of recruitment [46]. However, this may have been and under- or overestimate of the amount of cardiovascular morbidity present since some patients with CVD may not be taking any specific CVD drugs or may have stopped them (for example after myocardial infarction. Others may have been taking certain drugs as a preventative measure because of increased cardiovascular risk. Nonetheless our data show that patients on CVD drugs at baseline were significantly more likely to die from all-cause mortality, and heart disease in particular, than patients not on these drugs. Our data also show that patients taking CVD drugs at baseline had higher levels of MMP-2, 8 and 9 than patients not taking these drugs. However this does not explain the increased risk associated with elevated MMP-8 level, since this was associated with all-cause mortality independent of taking CVD drugs, and the latter were not associated with mortality from respiratory disease in models in which MMP-8 levels were highly significant.

A second possible limitation is the generalisability of the findings to the wider RA community since the patients studied were hospital-recruited and the study population was likely to include patients with more severe disease. The treatment of patients at baseline and during the follow up period also underwent changes in standard UK practice for treatment of hospital-based RA patients over the period 1993 to 2011. This would invariably have led to different treatment regimes based on the severity of disease at baseline, and during follow up. It is possible that different treatments may have resulted in different effects on MMP levels, but it was not possible to control for this in this study. Previous studies have indicated that the use of oral steroids is associated with increased morbidity and mortality from lower respiratory tract infections [12,47], and we have found in a separate, previously described population of well-characterised RA patients [48] that those taking oral steroids have significantly higher levels of MMP-8 and MMP-9 than those not taking these drugs (unpublished observations). We have also confirmed in this second population that MMP-8 levels are associated with mortality from pneumonia, but this was independent of steroid use, which did not demonstrate a significant association (unpublished observations).

The interpretation of death certificate data is another potential limitation to the current study, especially with regard to deaths from pneumonia or chest infections when the associated or contributing cause (for example, stroke) may in fact be the main cause. However, in the current study the majority of patients dying from pneumonia (*n *= 23/29) had this as the only cause, or the primary cause alongside RA as an associated cause. These patients also demonstrated a highly significant association of MMP-8 levels with mortality, similar to the total group who died from pneumonia (data not shown).

Another possible limitation was the measurement of MMP levels at a single time point, and cumulative measures of MMP-8 levels during follow up may possibly have provided better predictive information. Importantly the association of MMP-8 levels with mortality was independent of traditional measures of inflammation. In the present study, single measurements of ESR and CRP at study entry were not generally predictive of mortality in models that also contained MMP-8 levels.

## Conclusion

Our results indicate that in patients with established RA, high serum levels of MMP-8 are predictive of respiratory disease-related mortality, and provide additional predictive information on mortality in RA, which is not provided by traditional measures. These results also point to MMP-8 as a possible therapeutic target to reduce protease-mediated damage in pneumonia and other respiratory diseases in patients with RA.

## Abbreviations

BD: Becton Dickinson; CI: confidence intervals; COPD: chronic obstructive pulmonary disease; CRP: C-reactive protein; CVD: cardiovascular disease; DMARD: disease modifying anti-rheumatic drug; ESR: erythrocyte sedimentation rate; HAQ: health assessment questionnaire; HR: hazard rate; ICD: International Classification of Diseases; IQR: interquartile range; MMP: matrix metalloproteinase; NCSS: Number Cruncher Statistical System; NHS: National Health Service; National Health Service; NHSCR: NHS Central Register; ONS: Office for National Statistics; SD: standard deviation; RA: rheumatoid arthritis; RF: rheumatoid factor; VAS: visual analogue score.

## Competing interests

The authors declare that they have no competing interests.

## Authors' contributions

DLM and NBN carried out the biomarker measurements. DLM carried out the statistical analysis. NBN and PTD participated in the design of the study, and recruitment of patients. DLM conceived the study, participated in its design and coordination, carried out analysis and interpretation of data, and drafted the final manuscript. All authors read and approved the final manuscript.

## Supplementary Material

Additional file 1 **Table presenting correlations between levels of matrix metalloproteinases in patients with rheumatoid arthritis at baseline**. Click here for file

Additional file 2 **Table presenting correlations between clinical measures and levels of matrix metalloproteinases in patients with rheumatoid arthritis at baseline**. Click here for file
