# Supplementary material for: Association of circulating levels of MMP-8 with mortality from respiratory disease in patients with rheumatoid arthritis
Source: Arthritis Res Ther. 2012 Oct 2;14(5):R204. doi: 10.1186/ar4042 (PMC3580516; doi:10.1186/ar4042)
Supplement: Additional file 1 — Table presenting correlations between levels of matrix metalloproteinases in patients with rheumatoid arthritis at baseline. [file ar4042-S1.PDF]

Table S1. Correlation between serum MMP levels in RA patients at baseline

	MMP-2	MMP-3	MMP-8	MMP-9
MMP-1	0.056	**0.315**	**0.240**	**0.249**
MMP-2		0.074	-0.038	-0.081
MMP-3			**0.207**	**0.145**
MMP-8				**0.700**

Significant correlations (Spearman) are shown in bold.
