# Supplementary material for: Association of circulating levels of MMP-8 with mortality from respiratory disease in patients with rheumatoid arthritis
Source: Arthritis Res Ther. 2012 Oct 2;14(5):R204. doi: 10.1186/ar4042 (PMC3580516; doi:10.1186/ar4042)
Supplement: Additional file 2 — Table presenting correlations between clinical measures and levels of matrix metalloproteinases in patients with rheumatoid arthritis at baseline. [file ar4042-S2.PDF]

Table S2. Correlations between serum MMP levels and measures of inflammation and disease severity in RA patients at baseline

	Age	ESR	CRP	Larsen	Pain VAS	HAQ
MMP-1	**0.106**	**0.320**	**0.298**	**0.132**	**0.224**	**0.172**
MMP-2	**0.278**	-0.073	**-0.139**	0.007	**-0.128**	-0.040
MMP-3	**0.208**	**0.381**	**0.466**	**0.264**	**0.199**	**0.246**
MMP-8	-0.037	**0.233**	**0.230**	**0.144**	**0.107**	**0.097**
MMP-9	-0.001	**0.166**	**0.206**	**0.121**	0.050	0.089

Significant correlations (Spearman) are shown in bold.
